# Mitophagy-related long non-coding RNA signature predicts prognosis and drug response in Ovarian Cancer

**DOI:** 10.1186/s13048-023-01247-6

**Published:** 2023-08-26

**Authors:** Jiao Wang, Xiaocui Zhang, Fei Zheng, Qing Yang, Fangfang Bi

**Affiliations:** Department of Obstetrics and Gynecology, Shengjing Hospital of China Medical University, No. 36, Sanhao Street, Heping District, Shenyang, 110004 China

**Keywords:** Mitophagy, Long non-coding RNAs, LINC00174, Competitive endogenous RNAs, Ovarian cancer, Prognosis, Drug sensitivity

## Abstract

**Background:**

Ovarian cancer (OC) is the most malignant tumor with the worst prognosis in female reproductive system. Mitophagy and long non-coding RNAs (lncRNAs) play pivotal roles in tumorigenesis, development, and drug resistance. The effects of mitophagy-related lncRNAs on OC prognosis and therapeutic response remain unelucidated.

**Methods:**

We retrieved OC-related RNA sequence, copy number variation, somatic mutation, and clinicopathological information from The Cancer Genome Atlas database and mitophagy-related gene sets from the Reactome database. Pearson’s correlation analysis was used to distinguish mitophagy-related lncRNAs. A prognostic lncRNA signature was constructed using UniCox, LASSO, and forward stepwise regression analysis. Individuals with a risk score above or below the median were classified as high- or low-risk groups, respectively. The risk model was analyzed using the Kaplan–Meier estimator, receiver operating characteristic curve, decision curve analysis, and Cox regression analysis and validated using an internal dataset. LINC00174 was validated in clinical samples and OC cell lines. We also reviewed reports on the role of LINC00174 in cancer. Subsequently, a nomogram model was constructed. Furthermore, the Genomics of Drug Sensitivity in Cancer database was used to explore the relationship between the risk model and anti-tumor drug sensitivity. Gene set variation analysis was performed to assess potential differences in biological functions between the two groups. Finally, a lncRNA prognostic signature-related competing endogenous RNA (ceRNA) network was constructed.

**Results:**

The prognostic signature showed that patients in the high-risk group had a poorer prognosis. The nomogram exhibited satisfactory accuracy and predictive potential. LINC00174 mainly acts as an oncogene in cancer and is upregulated in OC; its knockdown inhibited the proliferation and migration, and promoted apoptosis of OC cells. High-risk patients were more insensitive to cisplatin and olaparib than low-risk patients. The ceRNA network may help explore the potential regulatory mechanisms of lncRNAs.

**Conclusion:**

The mitophagy-related lncRNA signature can help estimate the survival and drug sensitivity, the ceRNA network may provide novel therapeutic targets for patients with OC.

**Supplementary Information:**

The online version contains supplementary material available at 10.1186/s13048-023-01247-6.

## Background

Ovarian cancer (OC) is a heterogeneous tumor with the highest mortality rate and worst prognosis among gynecological malignancies [1]. There were 313,959 new cases and 207,252 deaths globally in 2020 [2]. The onset of OC is hidden. Patients often have no obvious symptoms in the early stage, and are often diagnosed when symptoms such as abdominal distension, abdominal pain, and weight loss appear in the late stage. Despite advances in diagnostic techniques and therapeutic strategies in recent years, the long-term survival of OC patients remains unsatisfactory [3]. Most patients with advanced-stage disease relapse and develop chemoresistance within a few years [4, 5]. Therefore, there is an urgent need to identify potential tumor prognostic markers and new therapeutic targets to guide treatment decisions and improve prognosis.

Mitophagy, the selective engulfment of dysfunctional or redundant mitochondria by autophagosomes and subsequent degradation in lysosomes, has been established as a major mechanism of mitochondrial quality control [6]. Abnormal mitophagy is associated with many diseases, including cardiovascular diseases [7], kidney disease [8], neurodegenerative disease [9], and cancer [10, 11]. The impact of mitophagy on cancer cells are multi-dimensional, and the specific role and mechanism of mitophagy in different cancers and at different cancer stages is still unclear [11]. The regulation of this process is critical for maintaining cellular homeostasis and has been implicated in acquired drug resistance [12]. In mammals, there are two main molecular regulatory mechanisms for mitophagy [13]: the Parkin-dependent pathway, involving PINK1/Parkin-mediated mitophagy; and the Parkin-independent pathway, in which mitophagy is mainly mediated by receptor proteins, such as BNIP3L/NIX, BNIP3, and FUNDC1 [11]. In addition, more and more new receptor molecules that can regulate mitophagy have been identified in recent years. A recent study showed that mitophagy is closely associated with cisplatin resistance in OC, and cisplatin resistance can be curtailed by blunting Bnip3-mediated mitophagy [14].

Long non-coding RNAs (lncRNAs) can bind to DNA, RNA, and proteins, and thus participate in gene regulation at the transcriptional, post-transcriptional and epigenetic levels [15]. LncRNAs are frequently dysregulated in cancer cells [16]; therefore, they can be considered as therapeutic, diagnostic and prognostic factors for cancer [15–17]. Thus, we explored the prognostic value of lncRNAs associated with mitophagy in OC and the possible regulatory mechanisms between lncRNAs and mitophagy-related genes. Our study may be valuable and meaningful for identifying potential prognostic markers and therapeutic targets in OC.

## Methods

### Data acquisition

RNA sequence, somatic mutation, and copy number variation (CNV) data, as well as clinicopathological information of 379 patients with OC, were downloaded from The Cancer Genome Atlas (TCGA) database. mRNA and lncRNA data were annotated using GTF files from Ensembl (http://asia.ensembl.org). We used Perl to integrate and extract lncRNA expression and corresponding clinicopathological data, including the patient number, age, stage, grade, survival status, and survival time. As reported in a previous article, we also used the Reactome database to obtain data on three mitophagy-related signaling pathways: PINK1/Parkin-mediated mitophagy (R-HSA-5,205,685), receptor-mediated mitophagy (R-HSA-8,934,903), and mitophagy (R-HSA-5,205,647) [18]. Based on the analysis of the combined gene set data, we identified 29 mitophagy-related genes (Additional file 1: Table S1). The “maftools” package in R software was used to present the mutation data of mitophagy-related genes.

### Identification of mitophagy-related lncRNAs

The correlation between lncRNAs and mitophagy-related genes extracted from the TCGA-OV dataset was calculated using the “corrplot” package, and mitophagy-related lncRNAs were screened out according to the p < 0.05 and |R| ≥ 0.4 screening criteria, using Pearson correlation analysis.

### Construction of the prognostic signature

Univariate Cox regression analysis was used to identify the mitophagy-related lncRNAs associated with the prognosis of patients with a setting of p < 0.05. LASSO Cox regression analysis with ten-fold cross-validation and forward stepwise regression analysis were then used to conduct a prediction signature of mitophagy-related lncRNAs. At the same time, the risk score of each patient was calculated using the following formula: risk score = Σ (expression value of each lncRNA × corresponding coefficient). The median risk score was used to classify patients into high- and low-risk groups.

### Validation of the prognostic signature

To validate this model, we performed a Kaplan Meier (KM) analysis to show the survival differences between the high- and low-risk groups and visualized the survival curves using the “survminer” and “survive” R packages. The 1-, 3-, and 5-year receiver operating characteristic (ROC) curves were drawn to evaluate prognostic prediction efficiency using the KM “survival ROC” R package. We performed univariate and multivariate Cox regression analyses to determine whether our risk model could independently predict prognosis in patients with OC. Furthermore, we divided the TCGA-OV dataset into two sets randomly, TCGA-training and -testing datasets, for internal validation of the model.

### Tissue collection

Samples of 51 OC and 40 normal ovarian tissues were collected from the tissue specimen Bank of Shengjing Hospital of China Medical University between 2015 and 2019. All OC patients had not received chemotherapy, radiotherapy or other antitumor therapy before surgery. This study was approved by the Ethics Committee of Shengjing Hospital of China Medical University, and informed consent was obtained from all patients.

### Reverse transcription-quantitative polymerase chain reaction (RT-qPCR)

Total RNA was extracted using TRIzol reagent (Takara Bio, Kusatsu, Japan) and evaluated using the NanoDrop 2000 system (Thermo Fisher Scientific, Carlsbad, CA, USA) to determine RNA purity and concentration. RNA samples were reverse transcribed using TransScript Uni All-in-One First-Strand cDNA Synthesis SuperMix for qPCR (One-Step gDNA Removal; TransGen Biotech, Beijing, China). PerfectStart Green qPCR SuperMix (TransGen Biotech) was used for qPCR using an ABI 7500 Fast System. The primer sequences of LINC00174 is as follows: forward: GGCCCAACACTTCCCTCAAA, reverse: CAGGGAGAAACGACCTGGAG. We used β-actin as an internal reference and the 2^−ΔΔCt^ method for gene expression analysis.

### Cell culture and transfection

We purchased cells from the Chinese Academy of Sciences Cell Bank (Shanghai, China) and cultured them in RPMI 1640 medium (Seven, Beijing, China) with 10% fetal bovine serum (FBS; Procell, Wuhan, China) in a 5% CO_2_ atmosphere at 37 °C. Long intergenic non-protein coding RNA 00174 (LINC00174) shRNA plasmid was purchased from GeneChem (Shanghai, China). We used lipofectamine 3000 (ThermoFisher Scientific, Waltham, MA) for transfection according to the manufacture’s protocol.

### Cell viability assay

The CCK-8 kit (GK10001, GLPBIO, Montclair, CA, USA) was used to assess the viability of cells. We inoculated the cell suspension in a 96-well plate (2 × 10^3^ cells/well), and added CCK-8 solution (10 µL per well) every 24 h, then incubated for 2 h. We measured the absorbance at 450 nm by using a microplate reader.

### Colony formation assay

We seeded 1000 cells per well of a 6-well plate. After 10 days in culture, we fixed and stained the cells with 4% paraformaldehyde (PFA) and 0.5% crystal violet, respectively; and then counted the colonies (> 50 cells).

### Cell scratch assay

We seeded cells in 6-well plates and waited for cells to grow to 90% confluency. We gently draw a straight line in each well with a 200 µL pipette tip, then washed the well 3 times with phosphate-buffered saline (PBS), and imaged the scratches with a microscope (Nikon, Japan) under 10x objective lens. Cells were cultured for 24 h in FBS-free medium before images were captured again.

### Transwell migration assay

Cell migration assay was performed using transwell chamber (8-µm pore size transwell filter) in a 24-well plate. We added 700 µL of medium containing 10% FBS to the lower chamber and 200 µL FBS-free medium (2 × 10^4^ cells) to the upper chamber. After incubation for 24 h, we fixed the cells with 4% PFA and stained the cells with 0.5% crystal violet. The stained migrated cells on the membrane were photographed under 20x objective lens and manually counted.

### Apoptosis assay with flow cytometry

After transfection for 48 h, the cells were collected and resuspended into a single cell suspension. Then we added Annexin V-FITC and PI staining solution (Vazyme, Nanjing, China) according to the manufacturer’s protocol. The cells were incubated in the dark for 10 min at room temperature and then analyzed by flow cytometry (Beckman Coulter, Brea, CA, USA).

### Construction of a nomogram model

The nomogram, which combined clinicopathological information and risk score, was plotted using the “rms” R package and analyzed to improve clinical applicability. Calibration curves were constructed to evaluate the consistency between the predicted and actual survival rates. Decision curve analysis (DCA) was used to incorporate patients or decision-makers preferences for clinical utility [19]. Meanwhile, area under the curve (AUC) values were calculated using the “survival,” “survminer,” and “time ROC” packages to compare the differential performance between the nomogram, risk score and clinicopathological information.

### Drug sensitivity analysis

We used the R package “pRRophetic” to predict the drug response. We used Ridge’s regression to estimate the half-maximum inhibitory concentration (IC50) of each patient, and 10-fold cross-validation to estimate the accuracy of the prediction. The drug sensitivity analysis was based on the Genomics of Drug Sensitivity in Cancer (GDSC) database [18].

### GSVA analysis and construction of a ceRNA Network

Gene set variation analysis (GSVA) is an unsupervised and nonparametric method for assessing the enrichment of gene sets associated with mRNA expression data [20]. We used the “GSVA” packages to assess the potential differences in biological functions between the high- and low-risk groups and constructed a ceRNA network based on selected mitophagy-related lncRNAs and corresponding mitophagy-related genes. The miRDB [21] and miRWalk [22] websites were used to identify microRNAs (miRNAs) interacting with selected mitophagy-related lncRNAs and mitophagy-related genes, respectively. The overlapping miRNAs were selected to construct the ceRNA network, which was visualized using Cytoscape software.

### Statistical analysis

Statistical analyses were performed using R (v4.0.0) and GraphPad Prism 9 (La Jolla, CA, USA). Univariate and multivariate Cox regression analyses were used to evaluate the independence of the mitophagy-related lncRNA signature in OC. For comparisons between the two groups, the unpaired Student’s t-test was used for variables with normal distribution. The Mann–Whitney U test was used to analyze variables with non-normal distribution. Analysis of variance (ANOVA) or the Kruskal–Wallis test was used to compare three or more groups. Statistical significance was set at p < 0.05 unless otherwise specified. * p < 0.05, ** p < 0.01, *** p < 0.001, and **** p < 0.0001.

## Results

### Landscape of mitophagy-related genes in TCGA-OV dataset

**Fig. 1:**
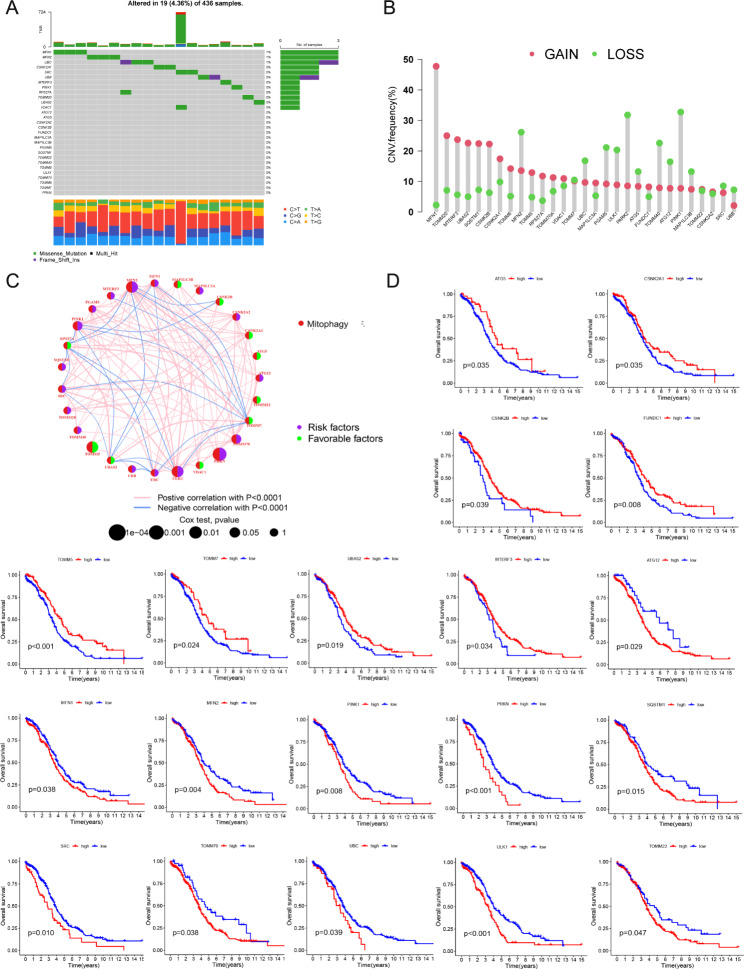
Landscape of mitophagy-related genes in TCGA-OV dataset. **A** The mutation frequency of 29 mitophagy-related genes. **B** The copy number variation condition. **C** The correlation network among the mitophagy-related genes (the circle size indicates the p value of the log-rank test, and the lines indicate the interactions between genes). **D** Prognostic value of 19 mitophagy-related genes in TCGA-OV dataset

Mutation analysis of 29 mitophagy-related genes in the TCGA-OV dataset indicated that 19 samples (4.36%) had gene mutations. The mutation frequencies in *MFN1*, *MFN2*, and *UBC* were the highest in OC patients (Fig. 1A). Common CNVs were identified in the 29 mitophagy-related genes, with *MFN1* having the maximum CNV amplification frequency and *PINK1* having the maximum CNV deletion frequency (Fig. 1B). Among the 29 mitophagy-related genes, the expression of *TOMM6* (translocase of outer mitochondrial membrane 6) was zero in OC samples in the TCGA-OV dataset, and thus not included in further analyses. Network analysis revealed that *MFN2* had the strongest positive correlation with *PINK1* (Fig. 1C). KM analysis showed that 19 mitophagy-related genes were associated with patient survival. *ATG5*, *CSNK2A1*, *CSNK2B*, *FUNDC1*, *TOMM5*, *TOMM7*, and *UBA52* were prognostic protective factors against overall survival (OS) in OC patients, whereas *ATG12*, *MFN1*, *MFN2*, *PINK1*, *PRKN*, *SQSTM1*, *SRC*, *TOMM70*, *UBC*, and *ULK1* were prognostic risk factors (Fig. 1D).

### Establishment of a prognostic risk model based on the mitophagy-related lncRNAs for OC patients

**Fig. 2:**
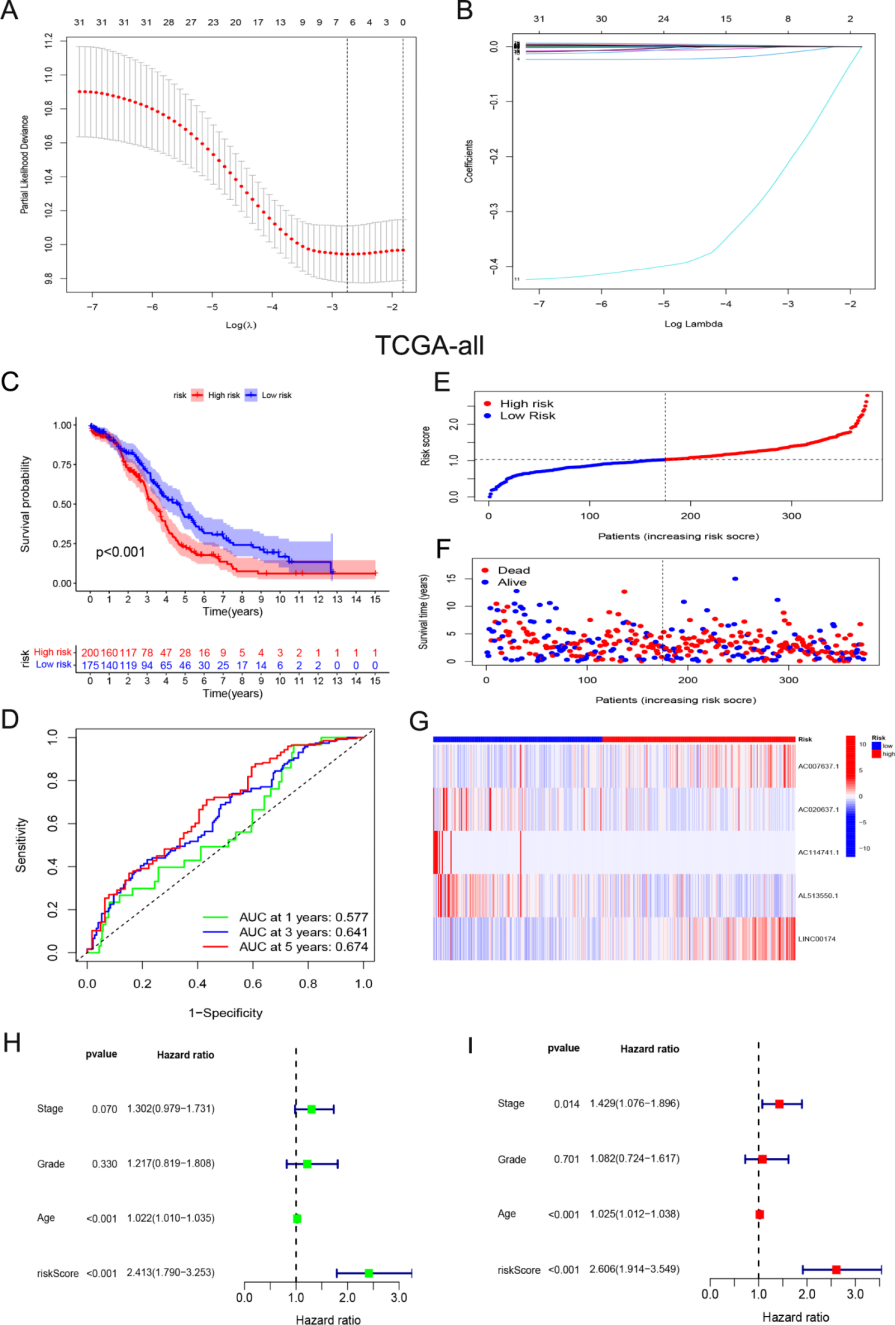
Construction of the prognostic model based on the TCGA-all dataset. **A, B** LASSO Cox regression analysis. **C, D** The KM curve and ROC curve for the risk model in predicting the OS of OC patients. **E, F** The risk score distribution and survival status of patients. **G** The heat map of the expression of 5 mitophagy-related lncRNAs. **H, I** Univariate and multivariate Cox analysis to assess the independence of the risk score

**Fig. 3:**
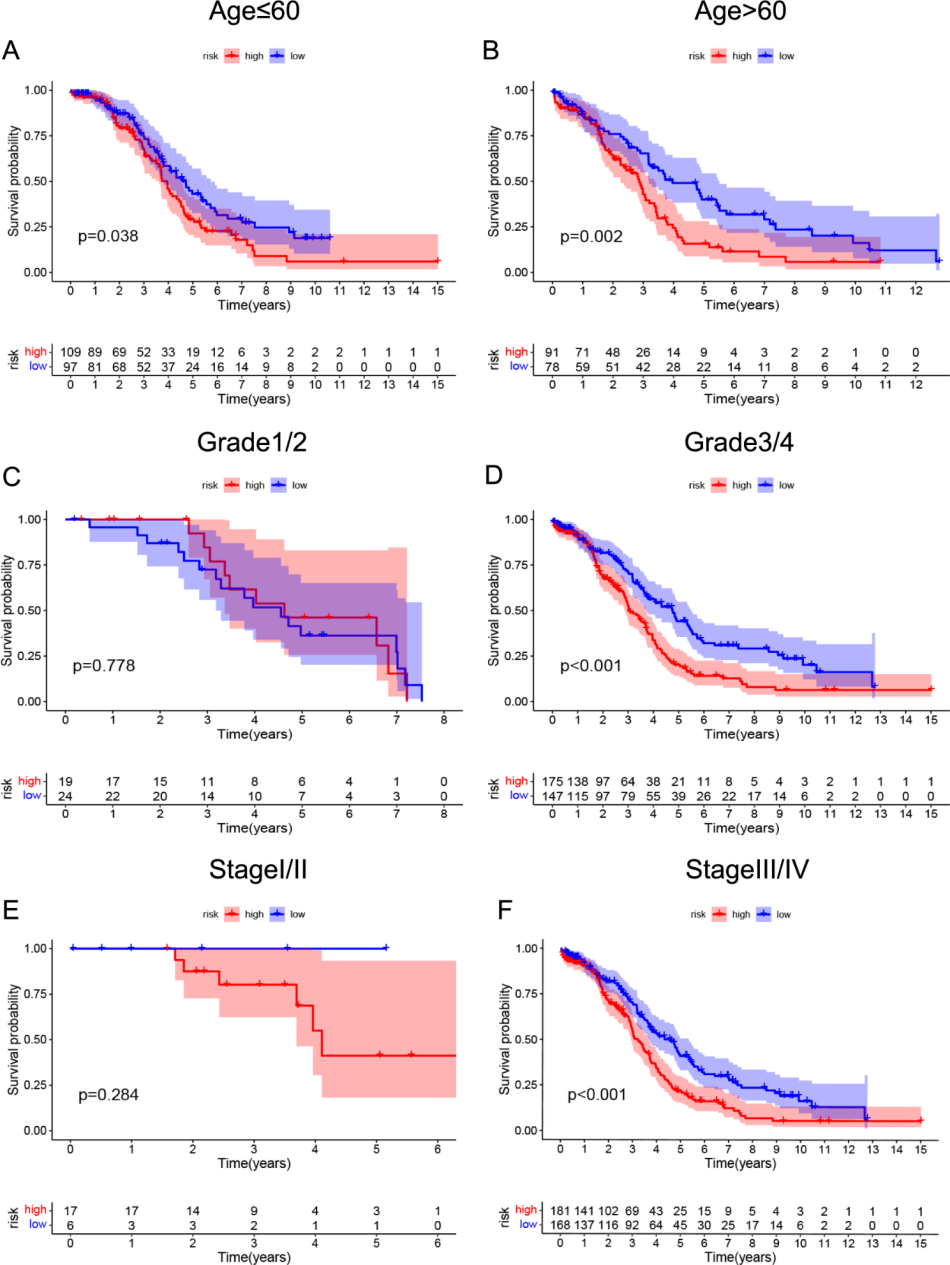
Stratified survival analysis in the TCGA-all dataset. **A-F** The high-risk group showed a poor prognosis than the low-risk group in different clinical stratification, except for the grade 1/2 and stage I/II groups

Pearson correlation analysis identified 331 lncRNAs that met the screening criteria (Additional file 2: Table S2). A network diagram of the 331 selected lncRNAs and 28 mitophagy-related genes is shown in Additional file 3: Fig. S1. Univariate Cox analysis was conducted to select 31 prognosis-related lncRNAs (selection criteria, p < 0.05), and the results are shown in Table 1. LASSO Cox regression and forward stepwise regression analyses were performed on the 31 prognosis-related lncRNAs (Fig. 2A, B). Finally, five lncRNAs (AC007637.1, AC020637.1, AC114741.1, AL513550.1, and LINC00174) were selected to establish a risk score model. The prognostic model formula we obtained to assess the risk score for each patient was as follows: risk score = 0.0035 × exp^AC007637.1^-0.023 × exp^AC020637.1^-0.3839 × exp^AC114741.1^-0.0011 × exp ^AL513550.1^+0.0008 × exp^LINC00174^. Patients were divided into high- and low-risk groups according to the median risk score. KM analysis of the curve demonstrated that patients in the high-risk group had shorter OS than those in the low-risk group (Fig. 2C). The ROC curve showed the specificity and sensitivity of the risk model for predicting the patient prognosis (Fig. 2D). The survival status of patients and the expression levels of five lncRNAs are shown from low to high risk (Fig. 2E–G). Univariate and multivariate Cox analyses showed that the model was an independent influencing factor in predicting the prognosis of patients (Fig. 2H, I). In addition, stratified prognostic analysis based on clinicopathological characteristics showed that, except for grade 1/2 and stage I/II groups, the prognosis of patients in the high-risk group remained poor (Fig. 3).

**Table 1:** The 31 mitophagy-related lncRNAs with a significant prognostic value

Id	HR	HR.95 L	HR.95 H	p value
AC007637.1	1.004387563	1.001773749	1.007008196	0.00099152
AC073046.1	1.000810794	1.00032226	1.001299567	0.001140439
SEMA6A-AS1	1.011022227	1.003971178	1.018122797	0.002141365
AL590729.1	1.00595448	1.002083848	1.009840062	0.002542013
AC005034.6	1.001220447	1.000394508	1.002047067	0.00377101
ACAP2-IT1	1.004787158	1.001524102	1.008060846	0.004007016
AL391335.1	1.007204349	1.002290243	1.012142548	0.004018574
LINC00174	1.0008445	1.000266835	1.001422498	0.004160958
AC073332.1	0.998190358	0.996899156	0.999483232	0.006094355
LINC02035	1.00126421	1.000301614	1.002227732	0.010039236
AL121944.2	0.999358132	0.998857385	0.99985913	0.012042886
AC245060.2	1.000803879	1.000168723	1.001439438	0.013108134
AL133230.2	1.011961706	1.001922967	1.022101027	0.019405698
AC131953.2	1.008978946	1.001311036	1.016705576	0.021643053
AC145423.3	1.003458336	1.000466158	1.006459463	0.023461274
AL450998.3	1.002731643	1.000327299	1.005141767	0.025938301
AC020637.1	0.973505795	0.95067642	0.996883391	0.026569837
AL513550.1	0.999235213	0.998559054	0.999911829	0.026741474
AC125494.1	1.012283541	1.001318404	1.023368755	0.028015712
ARF4-AS1	0.99826815	0.996722453	0.999816245	0.028350216
AC083806.2	1.014302692	1.001035496	1.027745725	0.034512788
Z94721.2	1.031102379	1.001976555	1.061074843	0.036168373
AC020916.2	1.001160631	1.000068291	1.002254164	0.037290602
YEATS2-AS1	1.003029456	1.000165072	1.005902042	0.038164271
AC074135.1	1.000474777	1.000023989	1.000925769	0.038990328
SEC 62-AS1	1.004040057	1.0001878	1.007907151	0.039810806
KDM2B-DT	1.002092434	1.000077782	1.004111145	0.041779093
AC022400.6	1.002499231	1.000083882	1.004920414	0.042548388
NRSN2-AS1	0.999725747	0.999460585	0.99999098	0.042702475
AC018521.6	1.001175291	1.000038	1.002313876	0.042817041
AC114741.1	0.713087734	0.512733105	0.991732564	0.044506036

### Validation of the prognostic risk model

**Fig. 4:**
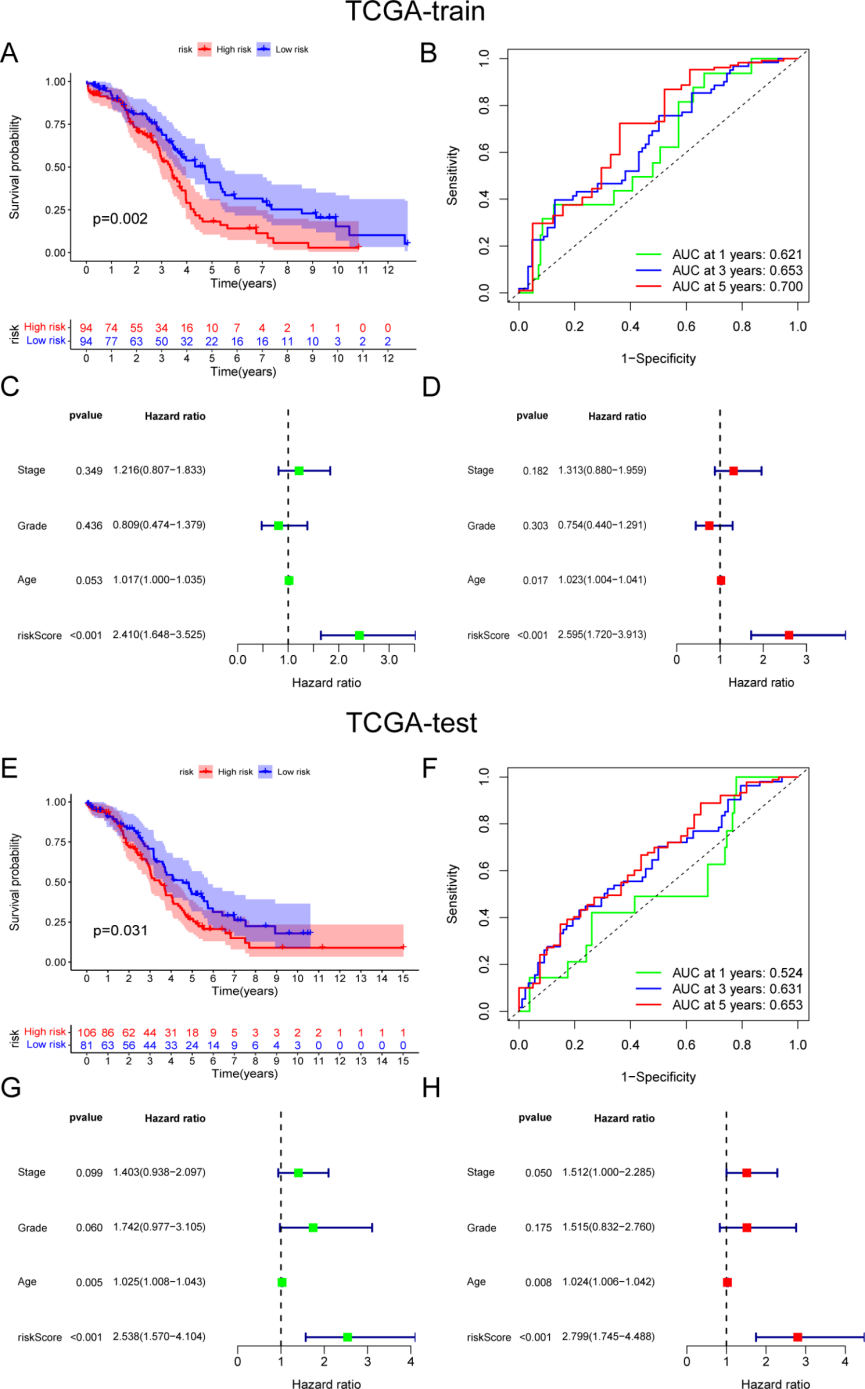
Internal validation of the prognostic model. **A** The KM curve for the risk score in predicting the OS of OC patients in the TCGA-training dataset. **B** ROC curve to show the sensitivity and specificity of the prognosis model in TCGA-training dataset. **C, D** Univariate and multivariate Cox analysis to assess the independence of the risk score in the TCGA-training dataset. **E** The KM curve for the risk score in predicting the OS of OC patients in the TCGA-testing dataset. **F** ROC curve to show the sensitivity and specificity of the prognosis model in TCGA-testing dataset. **G, H** Univariate and multivariate Cox analysis to assess the independence of the risk score in the TCGA-testing dataset

Patients in the TCGA-OV dataset were randomly divided into two datasets, TCGA-training and -testing, to confirm the performance of the risk model through internal validation. KM analysis indicated that the low-risk group patients lived longer in both the TCGA-training and -testing datasets (Fig. 4A and E). The ROC curve showed the specificity and sensitivity of the risk model in predicting the prognosis of patients in the TCGA-training and -testing datasets, and the AUCs for the 5-year OS were 0.700 and 0.653, respectively (Fig. 4B and F). Univariate and multivariate Cox analyses demonstrated that the risk score was an independent prognostic factor for OC patients in both the TCGA-training and -testing datasets (Fig. 4C, D, G, and H). In summary, by using the same formulation, we obtained consistent results for the training and testing cohorts, confirming the robustness of the risk model.

### LINC00174 is overexpressed in OC, and knockdown of LINC00174 inhibits proliferation and migration, and promotes apoptosis of OC cells in vitro

**Fig. 5:**
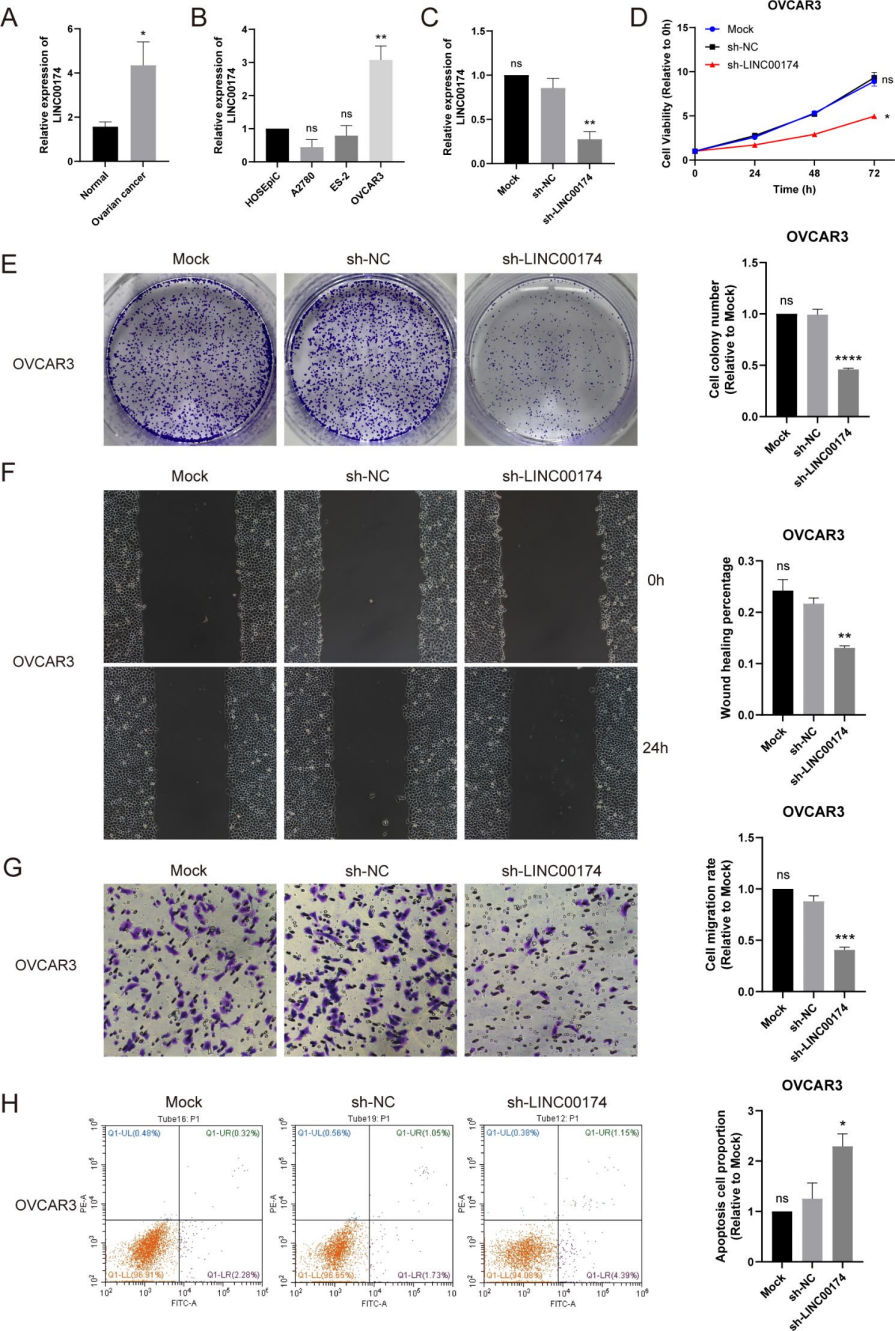
Experimental verification of LINC00174 in OC samples and cells. **A** Expression levels of LINC00174 in clinical samples (51 ovarian cancer and 40 normal ovarian tissues). **B** Expression levels of LINC00174 in normal ovarian epithelial cell line (HOSEpiC) and three OC cell lines (A2780, ES-2, and OVCAR3). **C** The knockdown efficiency of LINC00174 examined by RT-qPCR. **D-H** CCK-8 assay (**D**), colony formation assay (**E**), wound healing assay (**F**), transwell migration assay (**G**) and apoptosis assay (**H**) showed that knockdown of LINC00174 inhibited proliferation and migration, and promoted apoptosis of OVCAR3 cells

We first reviewed the role of LINC00174 in cancer in previous literature reports (Table 2) and observed that it mainly acts as an oncogene in cancer. As mentioned earlier, LINC00174 was highly expressed in the high-risk group and associated with poor prognosis in our risk model. qRT-PCR was performed to examine the expression of LINC00174 in 40 normal ovarian samples and 51 OC samples, revealing that LINC00174 expression was elevated in the OC samples (Fig. 5A). We then measured the expression of LINC00174 in a normal ovarian epithelial cell line (HOSEpiC) and three OC cell lines (A2780, ES-2, and OVCAR3), and observed that LINC00174 expression was significantly increased in OVCAR3 cells (Fig. 5B). Therefore, we chose OVCAR3 for subsequent cell function experiments. We downregulated LINC00174 expression in OVCAR3 cells using an shRNA plasmid. The knockdown efficiency was examined by RT-qPCR (Fig. 5C). CCK-8 assay results showed that LINC00174 knockdown decreased cell viability (Fig. 5D). Downregulation of LINC00174 also inhibited colony formation by OVCAR3 (Fig. 5E). Scratch and transwell assays revealed that the migration ability of OVCAR3 cells decreased after LINC00174 knockdown (Fig. 5F, G). Apoptosis assay showed that the proportion of apoptotic cells increased after LINC00174 knockdown (Fig. 5H).

**Table 2:** Overview of the role of LINC00174 in cancer

Cancer	Expression	Functions	Role	Mechanism	Reference
NSCLC	Downregulated	Overexpression of LINC00174 inhibited NSCLC cell proliferation and migration, and induced apoptosis	Tumor suppressor	LINC00174/miR-31-5p/LATS2 axis	[47]
CRC	Upregulated	Overexpression of LINC00174 promoted CRC cell viability, proliferation, migration, invasion and EMT	Oncogene	LINC00174/miR-3127-5p/E2F7 axis	[34]
KIRC	Upregulated	Overexpression of LINC00174 promoted KIRC cell viability, proliferation, migration and invasion	Oncogene	LINC00174/miR-612/FOXM1 axis	[36]
HCC	Upregulated	Overexpression of LINC00174 accelerated proliferation and metastasis of HCC cells while reduced apoptosis	Oncogene	LINC00174/miR-320/S100A10 axis	[37]
BCa	Upregulated	Knockdown of LINC00174 attenuated proliferative and migratory abilities in BCa cells.	Oncogene	LINC00174/miR-1827	[38]
Glioma	Upregulated	Knockdown of LINC00174 significantly suppressed GBM cells proliferation	Oncogene	-	[39]
OS	Upregulated	Knockdown of LINC00174 suppressed OS Cell Proliferation, Migration, Invasion and OS Tumor Growth	Oncogene	LINC00174/miR-378a-3p/SSH2 and TGF-β/SMAD pathway	[43]
TETs	Upregulated	Knockdown of LINC00174 reduced cell proliferation, migration, and lipid droplets accumulation in TET cells	Oncogene	LINC00174/miR-145-5p/SCD5 axis	[44]
Glioma	Upregulated	Knockdown of LINC00174 could remarkably prevent cell proliferation and promote cell apoptosis in both glioma cells and Temozolomide-resistant glioma cells	Oncogene	LINC00174/miR-138-5p/SOX9 axis	[40]
Glioma	Upregulated	Knockdown of LINC00174 increased BTB permeability and reduced the expression of the tight junction-related proteins ZO-1, occludin, and claudin-5	Oncogene	LINC00174/miR-138-5p (miR-150-5p)/FOSL2 feedback loop	[41]
Glioma	Upregulated	Knockdown of LINC00174 inhibited cell proliferation, migration, invasion and glycolysis of glioma cells	Oncogene	LINC00174/miR-152-3p/SLC2A1 axis	[42]
CRC	Upregulated	Knockdown of LINC00174 could repress CRC cell growth	Oncogene	LINC00174/miR-1910-3p/TAZ axis	[35]
LUSC	Upregulated	Knockdown of LINC00174 could repress LUSC cells proliferation, migration, and invasion while promoting cell apoptosis	Oncogene	LINC00174/miR-185-5p/NFIX axis	[45]
LC	Upregulated	Knockdown of LINC00174 could repress LC cells proliferation, migration, invasion and angiogenesis while promoting cell apoptosis	Oncogene	LINC00174/miR-584-3p/RPS24 axis	[46]

Abbreviations: NSCLC, non-small cell lung cancer; CRC, colorectal cancer; KIRC, kidney renal clear cell carcinoma; HCC, hepatocellular carcinoma; Bca, breast cancer; OS, osteosarcoma; TETs, thymic epithelial tumors; LUSC, lung squamous cell carcinoma; LC, lung cancer

### Construction of a nomogram model

**Fig. 6:**
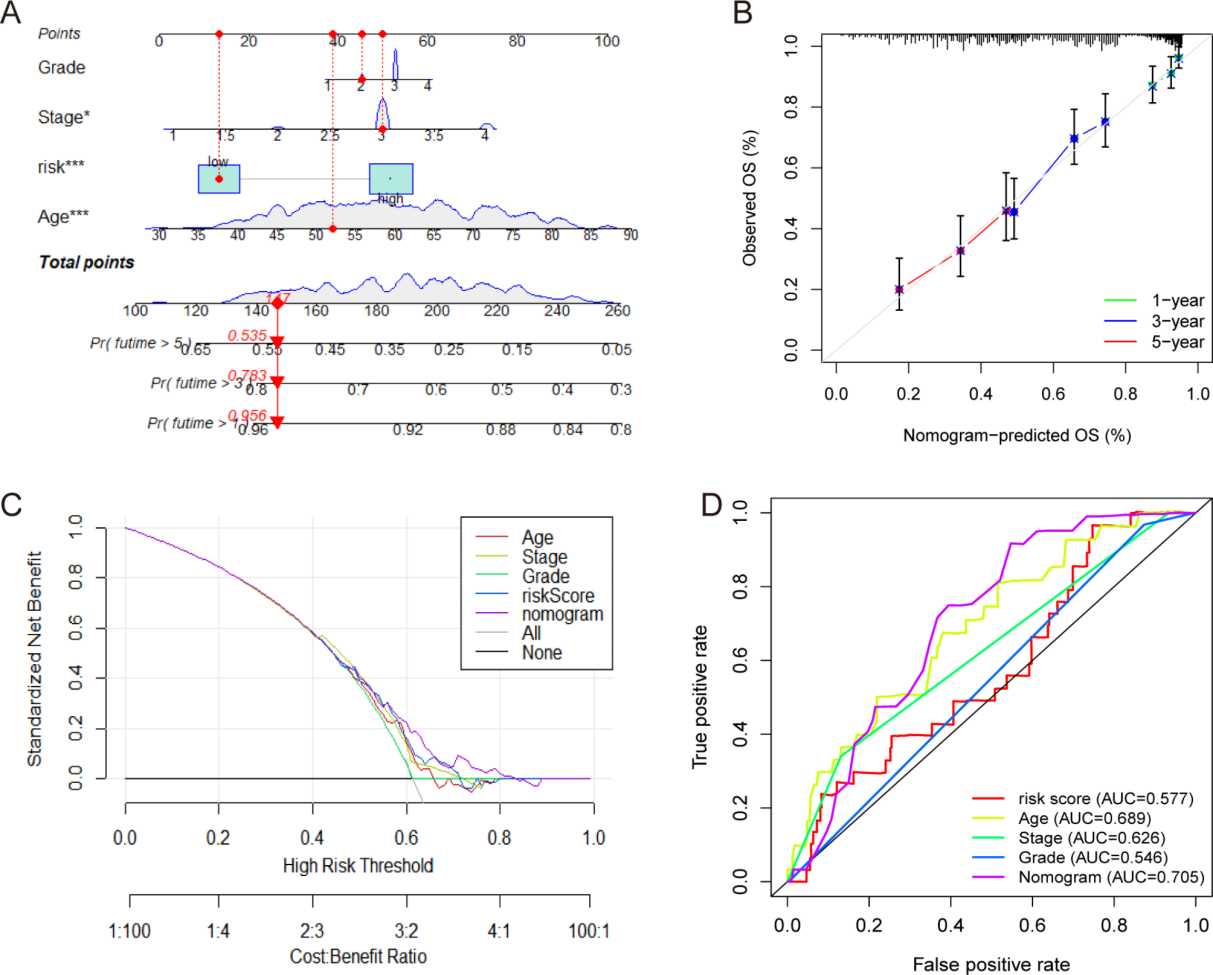
Construction of the nomogram model. **A** Nomogram for predicting the 1-, 3-, and 5-year OS of patients in the TCGA-OV dataset. **B** Calibration curves of the nomogram model of 1-, 3-, and 5-years. **C** DCA curves for predicting the OS of different parameters. **D** ROC curves for predicting the OS of different parameters

A nomogram model combining risk score, age, grade, and the stage was constructed to improve clinical applicability (Fig. 6A). The calibration curves for 1-, 3-, and 5-year OS indicated a high consistency between the actual observations and nomogram predictions (Fig. 6B). The DCA curve demonstrated that the nomogram model had the highest net benefit compared with the risk model and clinicopathological characteristics (Fig. 6C). The ROC curve showed that the nomogram model had the best sensitivity and specificity in predicting prognosis compared with the risk model and individual clinicopathological characteristics (Fig. 6D). These results demonstrate that the constructed nomogram is clinically practical for predicting the survival probability of patients with OC.

#### Drug sensitivity analysis

**Fig. 7:**
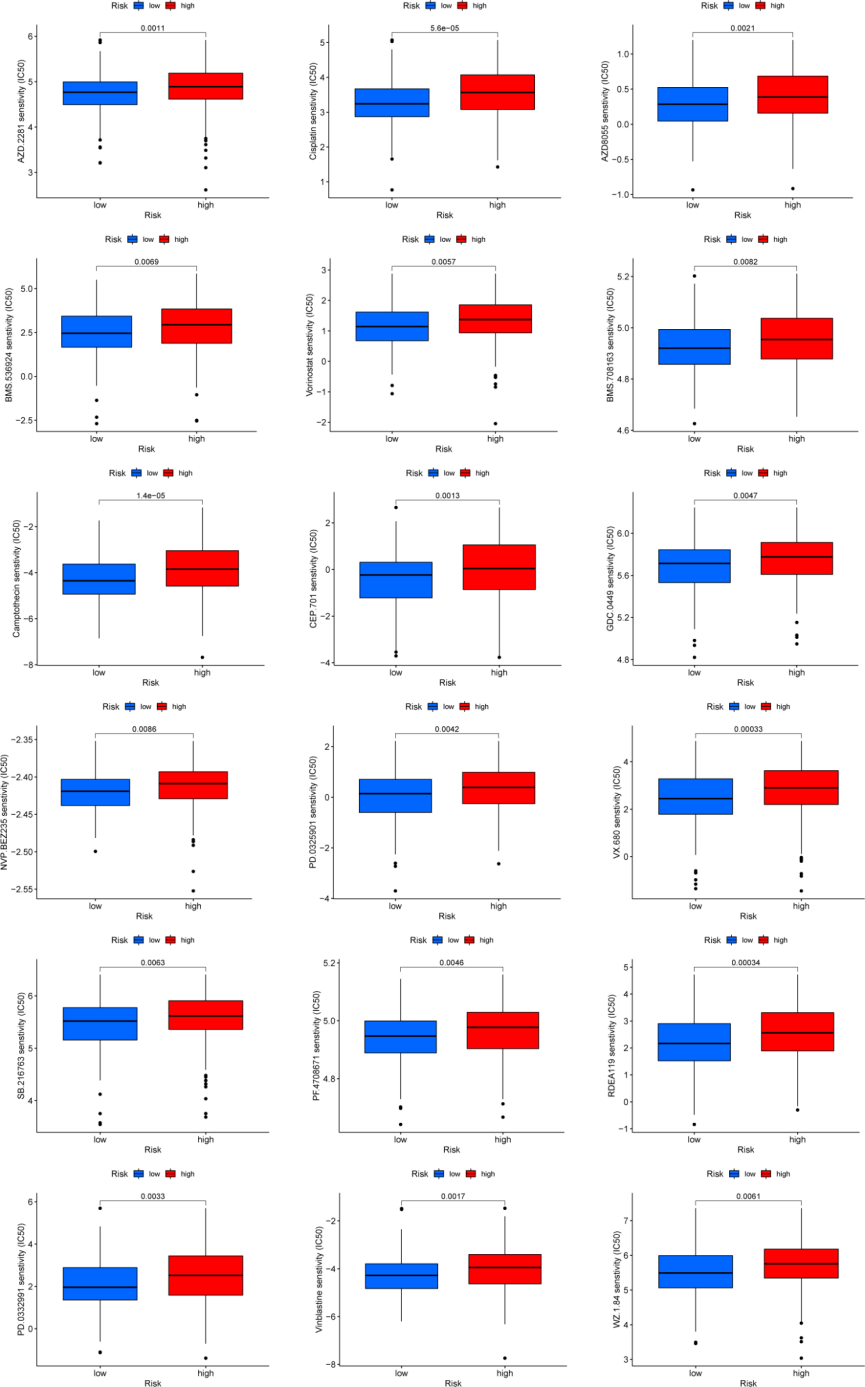
Drug sensitivity analysis. A total of 18 potential anti-tumor drugs with higher IC50 in the high-risk group

**Fig. 8:**
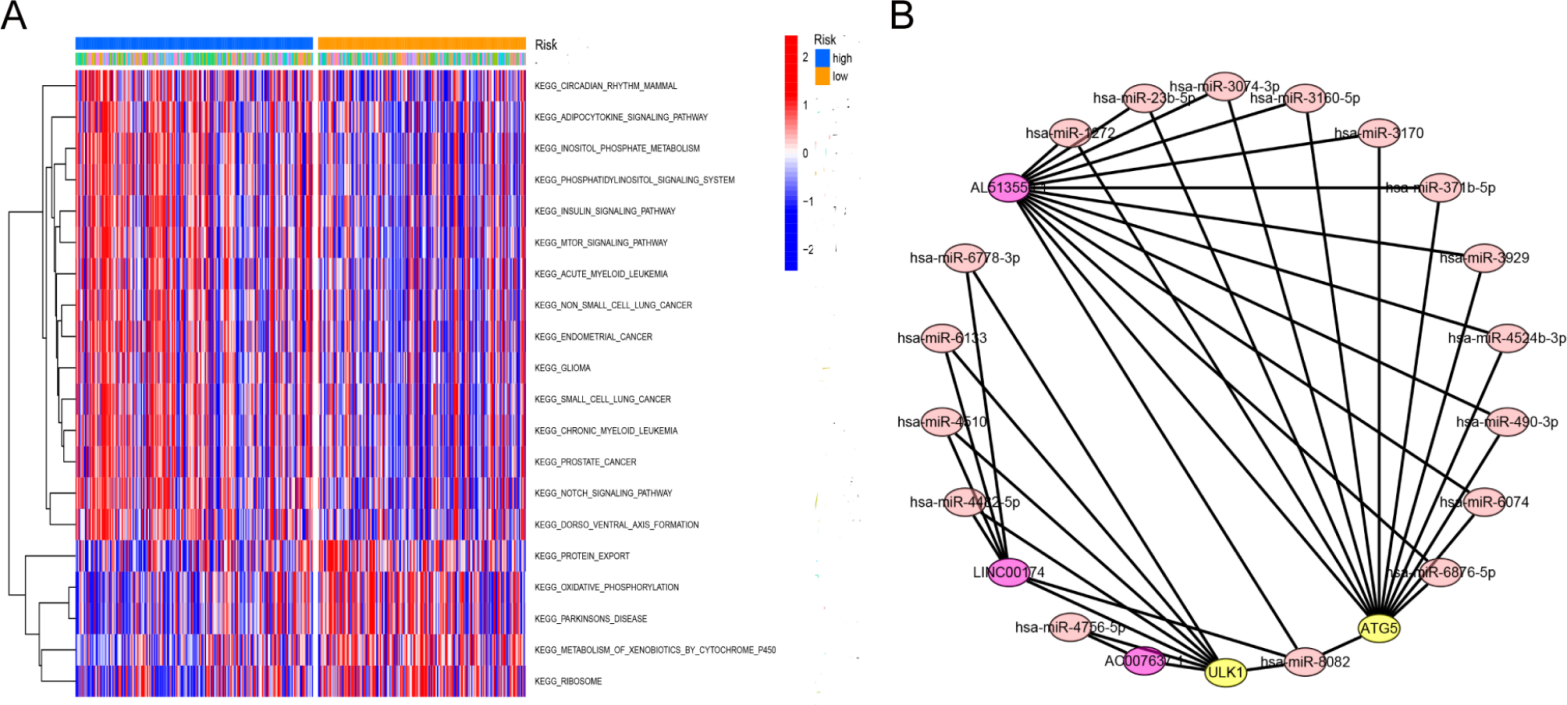
GSVA analysis and construction of a ceRNA network associated with the mitophagy-related lncRNAs. **A** GSVA enrichment analysis showed the activation states of biological pathways in high- and low-risk groups and the heat map was used to visualize these biological processes. **B** The ceRNA network included three mitophagy-related lncRNAs, 17 miRNAs, and two mitophagy-related genes

We also evaluated the risk model for OC pharmacotherapy. We discovered that the sensitivity to 40 anti-tumor drugs was strongly correlated with the risk score; the IC50 was higher for 18 anti-tumor drugs in the high-risk group (Figs. 7) and 22 anti-tumor drugs in the low-risk group (Additional file 4: Fig. S2). A high risk score was associated with a higher IC50 of chemotherapy drugs such as olaparib (AZD.2281), cisplatin, mechanistic target of rapamycin (mTOR) pathway inhibitor (AZD8055), insulin receptor and insulin-like growth factor-1 receptor inhibitor (BMS.536,924), avagacestat (BMS.708,163), camptothecin, lestaurtinib (CEP.701), vismodegib (GDC.0449), phosphoinositide 3-kinase-mTOR inhibitor (NVP.BEZ235), mirdametinib (PD.0325901), palbociclib (PD.0332991), p70 ribosomal S6 kinase inhibitor (PF.4,708,671), refametinib (RDEA119), glycogen synthase kinase-3 inhibitor (SB.216,763), vinblastine, vorinostat, aurora kinase inhibitor (VX.680), and epithelial and endothelial tyrosine kinase inhibitor (WZ.1.84) (Fig. 8). Higher estimated IC50 values were obtained in the high-risk group than in the low-risk group, indicating that a higher risk score could predict decreased sensitivity to these therapeutic drugs in patients with OC. Thus, the risk model may guide clinical drug treatment for patients with OC.

### Functional analysis of the prognostic risk model

GSVA was performed to compare the different biological functions between the two groups. The high-risk group was mainly enriched in pathways related to cancer, leukemia, MTOR_SIGNALING_PATHWAY, INSULIN_SIGNALING_PATHWAY, and NOTCH_SIGNALING_PATHWAY. In contrast, the low-risk group was mainly enriched in pathways associated with OXIDATIVE PHOSPHORYLATION, PARKINSONS DISEASE, and RIBOSOME (Fig. 8A). To further illustrate the possible mechanisms by which lncRNAs regulate mitophagy-related genes, we constructed a ceRNA network with three mitophagy-related lncRNAs, 17 miRNAs, and two mitophagy-related genes (Fig. 8B).

## Discussion

OC is the deadliest gynecological cancer. The current first-line treatment of OC includes cytoreductive surgery and platinum–taxane chemotherapy [23]. Following frontline treatment, tumor recurs in most patients with OC; the five-year survival rate is approximately 45% [23]. Chemoresistance is a significant hindrance to therapeutic efficacy in patients with OC [24]. Although angiogenesis inhibitor bevacizumab and poly (ADP-ribose) polymerase (PARP) inhibitors, such as olaparib and niraparib, have shown efficacy in prolonging progression-free survival (PFS) in recent years, they do not extend OS [25, 26], indicating the need for more effective therapy.

Mitophagy, as a key mitochondrial quality control mechanism [8], plays an important role in the process of carcinogenesis, including its progression and treatment; and serves as an important regulatory mechanism for maintaining intracellular and extracellular homeostasis [11]. Mitophagy is considered a double-edged sword in cancer. On the one hand, it can reduce oxidative stress by clearing dysfunctional or redundant mitochondria, which may prevent carcinogenesis; on the other hand, it may protect tumor cells from apoptosis or necrosis by helping cancer cells survive under stress, thereby promoting cancer progression. Overall, the molecular mechanisms involved in the regulation of mitophagy are diverse and complex, and involve crosstalk [11]. Recent studies have revealed that mitophagy plays an important role in OC, particularly in chemotherapy resistance [14, 27]. Therefore, a better understanding of the role of mitophagy in OC development and chemoresistance may provide new prognostic markers and targets for the clinical treatment of OC.

In our study, we investigated the mutation status of mitophagy-related genes in samples from the TCGA-OV dataset and observed that 4.36% of the samples harbored gene mutations. Of all the mitophagy-related genes examined in the OC patient samples, *MFN1*, *MFN2*, and *UBC* exhibited the highest mutation frequencies. *MFN1* and *MFN2* are GTPases essential for mitochondrial fusion [28], and *UBC* plays a key role in maintaining ubiquitin homeostasis [29]. Mitochondrial fusion, division, and ubiquitin homeostasis play pivotal roles in mitophagy. In addition, we noted that alterations in CNV were common among mitophagy-related genes. These alterations may be the main factor responsible for the disturbed expression of some mitophagy-related genes, especially those encoding PINK1 and MFN1. KM analysis showed that 19 mitophagy-related genes were associated with the prognosis of patients with OC. For example, *ATG5*, *FUNDC1*, and *TOMM5* were determined to be prognostic protective factors, whereas *ATG12*, *MFN1*, *MFN2*, *PINK1*, and *PRKN* were prognostic risk factors.

Recently, a close correlation between mitophagy-related genes and lncRNAs was reported [30–33], and their interactions can regulate the expression of target genes and cellular biological functions. However, the roles of mitophagy-related lncRNAs in OC remain unclear and require further investigation. In this study, based on five mitophagy-related lncRNAs, a prognostic risk model was constructed. Furthermore, the results showed that the risk model is a reliable prognostic indicator of OC and that high risk scores are indicators of poor prognosis. Moreover, the risk model was correlated with clinicopathological features such as age, stage, and grade.

One of the five mitophagy-related lncRNAs, LINC00174, is an lncRNA whose high expression was associated with poor prognosis in our risk model. LINC00174 plays an oncogenic role in several cancers, including colorectal cancer [34, 35], renal clear cell carcinoma [36], hepatocellular carcinoma [37], breast cancer [38], glioma [39–42], osteosarcoma [43], thymic epithelial tumors [44], and lung cancer [45, 46]. Contrary to these conclusions, Cheng et al. suggested that LINC00174 acts as a tumor suppressor gene in non-small cell lung cancer, and overexpression of LINC00174 inhibits NSCLC cell migration and proliferation, and induces apoptosis [47]. However, the role of LINC00174 in OC has not been reported; thus, it was selected for experimental verification. We observed that the expression of LINC00174 in OC tissue was higher than that in normal ovarian tissue. At the same time, we detected the expression of LINC00174 in three OC cell lines. In OVCAR3 cells, the expression of LINC00174 was significantly higher than that in the normal ovarian epithelial cell lines. In contrast, in the other two OC cell lines, there was no increase in its expression. Therefore, we knocked down LINC00174 in OVCAR3 cells to study its role in the development of OC and demonstrated that cell viability and proliferation of OVCAR3 cells were significantly inhibited, as well as cell migration ability, while cell apoptosis was promoted. These results prove that LINC00174 plays a cancer-promoting role in OC and may become a potential therapeutic target for patients with OC.

To further understand the clinical applicability of the risk score, a nomogram combining the risk score and clinicopathological information was constructed, which proved to be a feasible tool for predicting the survival probability of OC patients. The GDSC database provides access to explore association between risk score and clinical treatment [18]. We noted that the sensitivity to 40 anti-tumor drugs was closely related to the risk score, providing a promising prospect for individualized treatment of OC patients in clinical practice. A higher risk score could predict decreased sensitivity to therapeutic drugs such as cisplatin and olaparib in OC patients, indicating that a higher risk score may be associated with cisplatin resistance. Therefore, the risk score is an independent prognostic tool as well as triggers further consideration of the relationship between mitophagy-related lncRNAs and OC therapeutics.

GSVA results showed that the high- and low-risk groups were enriched in different signaling pathways, further explaining the heterogeneity between the two groups and their potentially different mechanisms. The ceRNA mechanism is a widely reported method by which lncRNAs regulate mRNA expression. Therefore, we predicted miRNAs that may interact with mitophagy-related lncRNAs, and miRNAs that may interact with mitophagy-related genes using online websites and then constructed a ceRNA network. We speculated that mitophagy-related lncRNAs, such as LINC00174, may regulate the expression of downstream mitophagy-related genes through a ceRNA mechanism, thereby affecting the progression of OC. However, further studies are required to validate these findings.

Our study had some limitations. First, we did not validate our model using an external dataset. Second, we did not experimentally validate all five lncRNAs to clarify the practical application of the model in clinical practice, and we did not conduct relevant experiments on mitophagy. We also constructed a ceRNA network to speculate on the possible regulatory mechanism between mitophagy-related lncRNAs and mitophagy-related genes; however, we did not experimentally validate the ceRNA network. Nonetheless, the present model was validated using internal datasets; therefore, the results are still reliable and acceptable.

In summary, to the best of our knowledge, there are no previous reports on the use of mitophagy-related lncRNAs to predict the prognosis of patients with OC. We successfully constructed and verified a risk model based on five mitophagy-related lncRNAs for OC and identified a key lncRNA, LINC00174, that may contribute to OC development. The results of our study may contribute to further understanding of the role of mitophagy-related lncRNAs in OC progression as well as drug treatment responses, which highlights the potential of this model in prognosis prediction and targeted therapy of OC. Well-designed experiments are needed in the future to further verify the reliability of the model and the molecular mechanisms by which LINC00174 promotes OC cell proliferation and migration.

### Electronic supplementary material

Below is the link to the electronic supplementary material.

**Additional file 1: Table S1.** 29 mitophagy-related genes.

**Additional file 2: Table S2.** 331 mitophagy-related lncRNAs.

**Additional file 3: Figure S1.** The network diagram of the 331 selected lncRNAs and 28 mitophagy-related genes.

**Additional file 4: Figure S2.** 22 anti-tumor drugs with a higher IC50 in the low-risk group.

## Data Availability

Any reasonable requests for access to available data underlying the results reported in this article will be considered. Such proposals should be submitted to the corresponding author.

## Abbreviations

AUC: Area under curve
CNV: Copy number variation
ceRNA: Competing endogenous RNA
DCA: Decision curve analysis
FBS: Fetal bovine serum
GDSC: Genomics of Drug Sensitivity in Cancer
GSVA: Gene set variation analysis
IC50: Half-maximum inhibitory concentration
LINC00174: Long intergenic non-protein coding RNA 00174
lncRNAs: Long non-coding RNAs
OC: Ovarian cancer
OS: Overall survival
PARP: Poly (ADP-ribose) polymerase
PFA: Paraformaldehyde
PFS: Progression-free survival
ROC: Receiver operating characteristic
RT-qPCR: Reverse transcription-quantitative polymerase chain reaction
TCGA: The Cancer Genome Atlas
