# Supplementary figures and images for: Mitophagy-related long non-coding RNA signature predicts prognosis and drug response in Ovarian Cancer
Source: J Ovarian Res. 2023 Aug 26;16:177. doi: 10.1186/s13048-023-01247-6 (PMC10463594; doi:10.1186/s13048-023-01247-6)

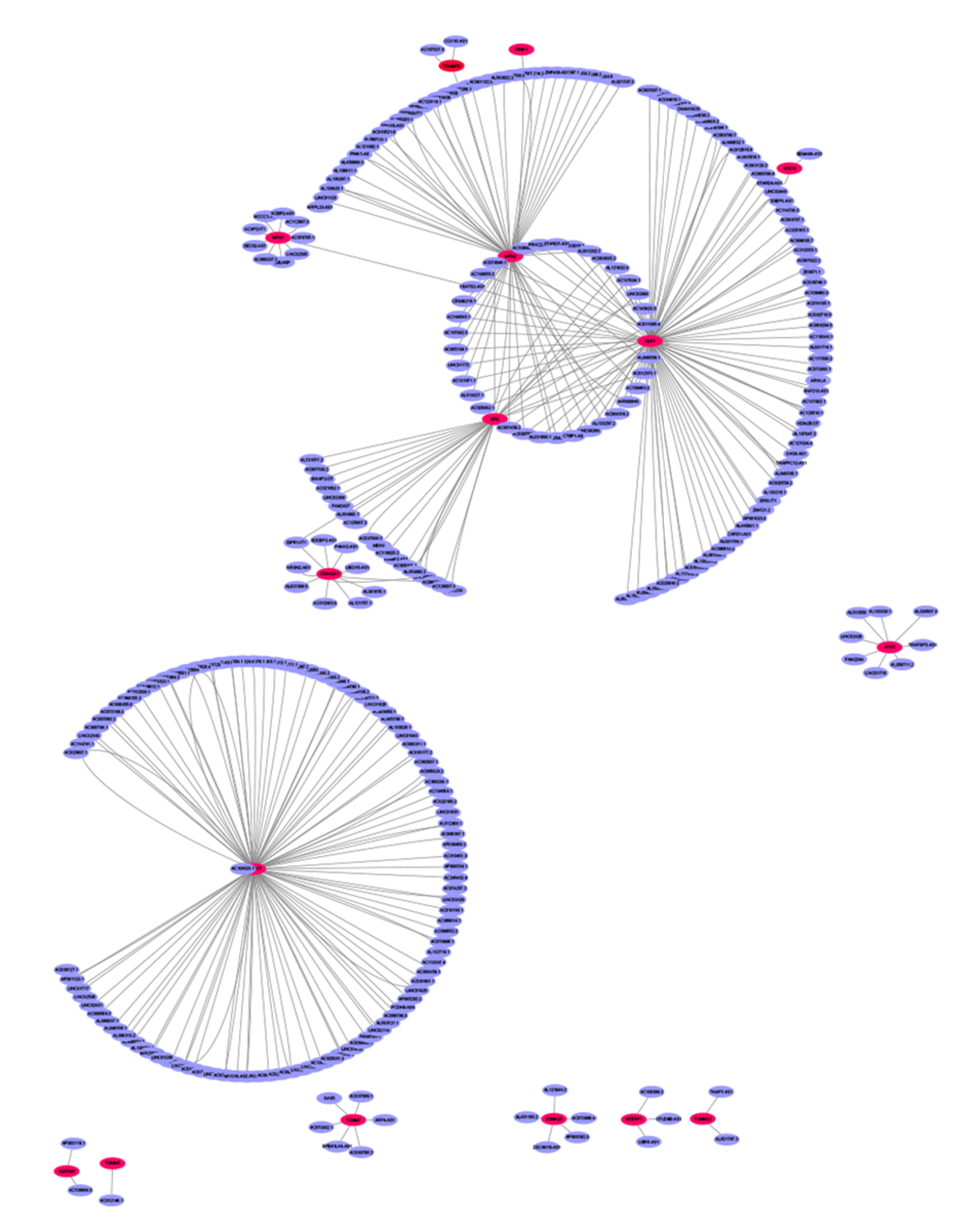

Supplement: Supplementary file 1 — Additional file 1: Table S1. 29 mitophagy-related genes. [file 13048_2023_1247_MOESM1_ESM.tif]

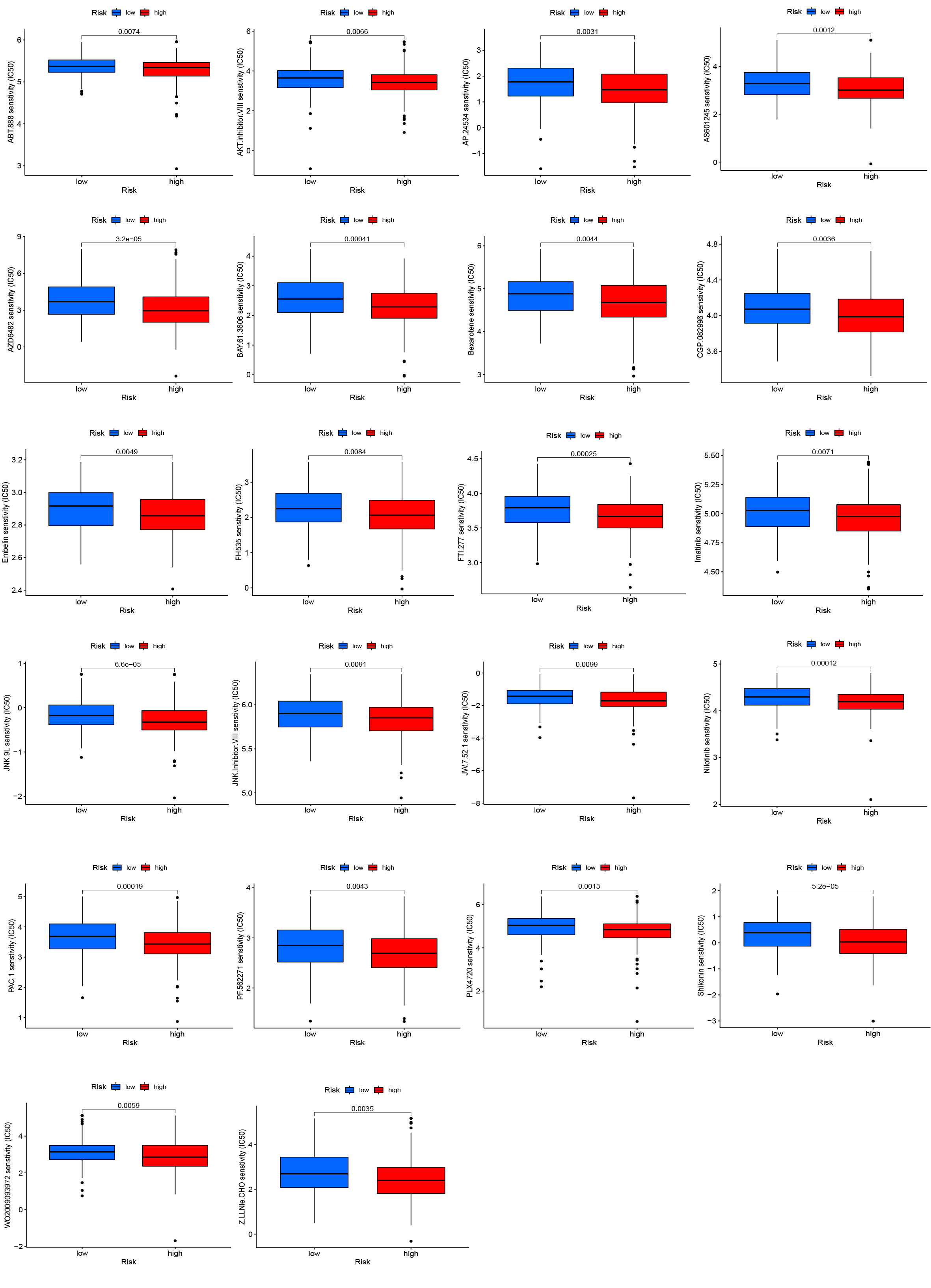

Supplement: Supplementary file 2 — Additional file 2: Table S2. 331 mitophagy-related lncRNAs. [file 13048_2023_1247_MOESM2_ESM.tif]
